# Alterations to the middle cerebral artery of the hypertensive-arthritic rat model potentiates intracerebral hemorrhage

**DOI:** 10.7717/peerj.2608

**Published:** 2016-11-03

**Authors:** Amy Randell, Killol Chokshi, Brittany Kane, Hilary Chang, Safaa Naiel, Jeffrey G. Dickhout, Noriko Daneshtalab

**Affiliations:** 1 School of Pharmacy, Memorial University of Newfoundland, St. John’s, Newfoundland and Labrador, Canada; 2 Department of Medicine, Division of Nephrology, McMaster University, Hamilton, Ontario, Canada

**Keywords:** Hypertension, Spontaneously hypertensive rats, Adjuvant-induced-arthritis, Hemorrhagic stroke, High salt diet, Neuro-inflammation, Pressure dependent constriction, Middle cerebral artery contraction, Intracerebral hemorrhage

## Abstract

**Aims:**

We have recently created an age-dependent hypertensive-mono-arthritic animal model from the stroke-resistant spontaneously hypertensive rat to model populations with autoimmune disease who are hypertensive and are prone to stroke. The model exhibits signs of hemorrhagic stroke (HS) subsequent to chronic inflammation and hypertension. HS is also associated with the inability of middle cerebral arteries to undergo pressure dependent constriction (PDC). We investigated alterations in the cerebrovasculature of our hypertensive mono-arthritic animals that develop stroke.

**Main Methods:**

Animals were fed either a high salt diet (HSD) (4% NaCl) or Purina chow (0.58% NaCl) from weaning. Complete Freund’s Adjuvant (CFA) was injected into the left hind paw at 21–28 weeks; controls received saline and histological and functional studies were performed.

**Results:**

Brain damage was more prominent with the high salt, with inflammation exacerbating the damage. High salt alone significantly decreased middle cerebral artery’s (MCA’s) ability to undergo PDC. Inflammation significantly decreased the ability of cerebrovasculature to respond to pressure step in the regular salt diet. The responses to vasoactive peptides were also significantly attenuated in both inflamed groups regardless of diet.

**Conclusion:**

Induction of chronic systemic inflammation increases brain damage, and affect the MCA’s vasogenic function, decreasing its ability to respond to intraluminal pressure. HSD further exacerbates organ damage associated with chronic inflammation, further compromising cerebrovascular function, and likely increasing the incidence of intracerebral hemorrhage and injury.

## Introduction

Autoimmune diseases such as rheumatoid arthritis (RA) are a chronically progressive inflammatory disease, with the leading cause of death being due to cardiovascular (CV) complications rather than the arthritis itself (Solomon et al., 2003; Gonzalez et al., 2008). Fundamental studies indicate significant risk of stroke in autoimmune arthritis, with patients with RA having a 30% increase in stroke over age-matched controls (Lindhardsen et al., 2012; Zöller et al., 2012). The risk of death from the first incidence of stroke has also been shown to be significantly higher for RA patients compared to non-arthritic subjects (Solomon et al., 2003; Book, Saxne & Jacobsson, 2005; Sokka, Abelson & Pincus, 2008). Of all stroke subtypes, hemorrhagic stroke (HS) has the highest mortality rate, approaching 50% within the first month (Thrift et al., 1996; Donnan et al., 2008), and is characterized by cerebral blood flow alteration.

Cerebral blood flow autoregulation is governed by a variety of physical cues (including sheer stress, pressure, hypoxia) and endogenous chemical stimuli (such as peptides, and cytokines) (Bassenge & Heusch, 1990; Vanhoutte & Mombouli, 1996). Pressure dependent constriction (PDC) in middle cerebral artery (MCA) ensures adequate and controlled perfusion of the small arteries feeding the brain (Johnson, 1986). Studies in stroke-prone spontaneously hypertensive rats (SHRsp) have revealed that MCAs ability to undergo pressure induced constriction is lost after HS (Smeda, 1992). The MCAs from post-stroke SHRsps have also lost the ability to respond to endothelial-mediated responses to various peptides such as bradykinin and nitric oxide synthase (NOS) inhibitors. Responses to vascular smooth muscle-mediated protein kinase C (PKC) activation (with phorbol dibutyrate) and intracellular calcium release (with vasopressin) are also attenuated (Smeda & King, 2000; Daneshtalab & Smeda, 2010). Although chronic hypertension and chronic inflammation have both been individually correlated to vascular dysfunction (Bassenge & Heusch, 1990; Sutherland & Auer, 2006; Sprague & Khalil, 2009), the impact of these factors in conjunction with the effectiveness and efficiency of MCA function has not yet been investigated.

We have recently established a model of aged spontaneously hypertensive rats (SHR) that are originally stroke-resistant, but which, upon induction of adjuvant-induced arthritis, develop severe signs of intracerebral hemorrhage. We also tested the effect of 4% NaCl on hemorrhage development and observed that high salt diet (HSD) increases intracerebral hemorrhage severity (Randell & Daneshtalab, 2016). The present study is designed to describe the impact of chronic inflammation, concurrent hypertension, and HSD on the ability of the MCA to respond to intraluminal pressure and to vasoactive peptides, and investigate its impact on degree cerebral damage. We believe the physiological conditions manifested by this animal model will help illuminate the mechanisms of cerebrovascular function of patients suffering from both RA and hypertension and lead to better understanding for treatment options to minimize fatal stroke.

## Materials and Methods

### Animals

All experimental procedures and animal breeding was carried out at Memorial University of Newfoundland Animal Care Facility and were in compliance with guidelines and recommendations set forth by the Animal Care ethics committee (Protocol #15-30-ND) and the Canadian Council on Animal Care (Guide to Care and Use of Experimental Animals, vol. 1, 2nd ed.). In total, 45 male Stroke resistant SHE (Original stock from Charles River Laboratories, Quebec, Canada) were included in the study. The animals were bred in-house and were housed two per cage in ventilated cages under standard light cycle (12 h light/dark), controlled temperature, and humidity conditions. Experimental design was implemented at 21–28 weeks of age. *Ad libitum* access to food and water was permitted.

### Experimental design

SHR-high salt diet (SHR-HSD) groups were fed a Japanese-style HSD containing 4% NaCl (Zeigler Bros, Gardners, PA, USA) from weaning. SHR-regular diet (SHR-RD) groups were maintained on standard rat chow (Laboratory Rodent Diet 500I; Lab Diet, St. Louis, MO, USA; 0.58% NaCl). At 21–28 weeks of age, they were randomly divided into four groups based on diet (HSD or RD) and treatment (Complete Freund’s Adjuvant (CFA) model of mono-arthritis or Saline (SAL) injected control). They were labelled SHR-HSD-SAL (n = 10), SHR-HSD-CFA (n = 14); SHR-RD-SAL (n = 11), SHR-RD-CFA (n = 10). Procedures for arthritis induction, weekly monitoring, and results are all described and published earlier (Randell & Daneshtalab, 2016). Briefly, the animals were anesthetized (isoflurane; Sigma), and either CFA (0.07 ml of 700 μg *M. butyricum*) or sterile 0.9% saline solution (0.07 ml) was injected (intradermal) into the plantar surface of the left hind paw of 21–28 week old SHRs at day 0 of the experimental procedure and animals were maintained for 21 days.

### Sample isolation and tissue processing

Necropsy was performed on day 21 after deep anesthesia (50/10 mg/kg of Ketamine:Xylazine) and exsanguination. The brain was removed and placed in oxygenated ice-cooled (≈3 °C) HEPES Bicarbonate Buffer (pH 7.4). The right and left MCAs were isolated, starting at the point distal to where it crosses the rhinalis fissure and mounted on a pressure myograph. The rest of the brain were immediately fixed in a 10% Neutral Buffered Formalin (Thermo Fisher) for further processing.

### Tissue preparation for histological assessment and immunofluorescent analysis

Upon fixation, samples were embedded in paraffin, and 6 μm cryosections were cut. Samples were stained using hemotoxylin and eosin (H&E) by standard procedures. Brain sections were also stained for astrocytes and microglia/macrophages in the cortex using glial fibrillary acidic protein (GFAP)-Cy3 (1:1,000; Sigma Aldrich, Montreal, CAN) and ionized calcium binding adaptor molecule 1(Iba-1; 1:1,000, Wako Chemicals, Richmond, VA, USA). Secondary antibody of Cy2 Goat Anti-rabbit (1:200; Jackson Immunoresearch, West Grove, PA, USA) and 4′,6-diamidino-2-phenylindole (DAPI) (1:1,000; Invitrogen, Hamilton, ON, USA) were applied in the second day according to established protocol. Stacks of images with 1 μm increments in a total of six slices in depth were collected with a confocal microscope (FV500; Olympus) with FluoView (Olympus) software.

### Quantification of neural damage

Neural and brain damage associated with the treatments were analyzed using the H&E stains of the samples, and scored using a scheme based on combinative semi-quantitative scoring system as outlined in recent research (Fedchenko & Reifenrath, 2014). To maximize detection and repeatability of the scoring system, our scoring contains four score levels (Shackelford et al., 2002). Table 1 outlines the grading scheme associated with each parameter scored. Cell vacuolation and neuron degeneration determine axonal swelling and associated cell death in the white matter, which occurs spontaneously or via a wide range of stimuli (Henics & Wheatley, 1999). The grading scheme for this cell death are valued points from 0 to 10, where cell count is graded in point system. The vacuolation is graded in factor of 10 cells per point, with the maximum number of vacuolation quantifiable being 80. The degenerating neurons were graded in factor of two cells per point, and the grading system from 0–10 was sufficient as the maximum number of degenerating neuron quantifiable was eight. Areas of edema and areas of cell infiltration (also indicators of brain injury and damage) were quantified separately, and graded in a factor of 10% for each point, the total being the complete image area. The grading scheme for the areas is a modified version adopted from the “quickscore” system (Detre, Saclani Jotti & Dowsett, 1995), which assigns values from 1 to 6 in proportion and multiplication is recommended instead of addition for processing of final score range. A total final score was determined via summation of the four separate parameters to indicate total damage evident in the samples.

**Table 1:** Scoring scheme for quantifying brain damage. Scoring system for four semi-quantifiable parameters (Cell vacuolation, degenerating neurons, area of cell oedema and area of cell infiltration). Total final score is the combination of A+B+C+D; it is the total of the score assigned for cell vacuolation, neuron degeneration, area of oedema & area of cell infiltration. A High final score indicates higher brain damage. The data obtained from the images with total magnification of 200× were used to determine the final score for each sample.

Score assigned	Number of cells undergoing vacuolation (A)	Number of degenerating neurons (B)	Percentage of area of oedema (C) (%)	Percentage of area of cell infiltration (D) (%)
0	0	0	0	0
1	1–10	1–2	1–10	1–10
2	11–20	3–4	11–20	11–20
3	21–30	5–6	21–30	21–30
4	31–40	7–8	31–40	31–40
5	41–50	9–10	41–50	41–50
6	51–60	11–12	51–60	51–60
7	61–70	13–14	61–70	61–70
8	71–80	15–16	71–80	71–80
9	81–90	17–18	81–90	81–90
10	91–100	19–20	91–100	91–100

### Quantification of Iba-1 and GFAP staining

Confocal images were analyzed with ImageJ software (National Institutes of Health, ImageJ1.40) and FIJI plugin. The images stained for astrocytes (GFAP), microglia (Iba-1) & nucleus (DAPI) were calibrated and gray scaled. Mean integrated density, area of measurement, background integrated density was measured for astrocytes. We used Corrected total tissue fluorescence (CTTF), where CTTF = {(MID) × (TA)} − BMID; MID is the mean integrated density, TA is the total area of measurement, BMID is the background mean integrated density, with the unit being pixels per μm. Triplicate reading of background intensity per image were obtained and the mean was used for final calculation.

The quantification of microglia was based on cell count and type of microglia. Microglia undergo transformation with injury and are activated. They adopt amoeboid shape with very short projections. The amoeboid shaped cells that were counted as activated microglia. The microglia which did not undergo transformation were labelled as type 2 cells. The total detectable microglia cells over DAPI Stained nuclei was used for the final analysis.

### Pressure myograph experiments

All chemicals used in the pressure myograph experiments were purchased from Sigma Aldrich (Montreal, CAN) unless otherwise stated.

Isolated MCAs were mounted onto the Single Vessel Chamber component of the Pressure Servo System (Living Systems Instrumentation, St Albans City, VT, USA) for pressure myograph studies. Vascular response was imaged using an inverted microscope and measured using a Video Dimension Analyzer (Living Systems Instrumentation, VT, USA). Mounted vessels were tied off creating a blind sack and pressurized to 100 mmHg and equilibrated for 30–45 min in an oxygenated (95:5% O_2_:CO_2_) HEPES bicarbonate buffer (NaCl, 130 mM; KCl, 4 mM; MgSO_4_, 1.2 mM; NaHCO_3_, 4 mM; CaCl_2_, 1.8 mM; HEPES 9.9 mM; KH_2_PO_4_, 1 mM; EDTA, 2.4 M; Glucose, 5.8 mM) in a temperature controlled (37 °C) environment. Baumbach & Heistad (1989) previously described a decrease in blood pressure (BP) of > 50% between that measured in the femoral or carotid artery compared to that measured in the distal portions of the middle cerebral vessels (Baumbach & Hajdu, 1993; Baumbach, Faraci & Heistad, 1994). As a result, all their pressure myograph experiments were conducted at a resting pressure of 100 mmHg. We also conducted our myograph experiments in the MCA at resting pressure of 100 mmHg, as we believe the setting accurately models physiological mean BP in vivo in the MCA of the SHR, as arterial systolic BP ranges from 200 to 230 mmHg (Yamori, 1984). PDC was evaluated first. Following equilibration, the pressure was decreased to 0 mmHg for 6 min to disengage PDC (Smeda & King, 2000). After this resting period, the pressure was immediately reapplied to 100 mmHg and lumen diameter was recorded (at the instant when the vessel experienced maximal pressure-mediated dilation; t = 0). The ability of the artery to constrict to pressure was determined in the change in MCA lumen diameter between 1 s to 6 min after the reapplication of pressure. The percentage decrease in lumen diameter observed in response to the latter pressure step in the MCAs was calculated to be PDC. The bradykinin (1.6 μM) mediated response was tested by measuring the maximal vasodilatory response between 15 s to 2 min, and were assessed at 100 mmHg pressure. Results were expressed as a percentage of maximal relaxation produced by nifedipine (3 μM). Maximal responses observed within 15 s, were followed by reconstriction to a Lumen Diameter (LD) comparable to that present prior to the application of the peptide.

After the preparation was flushed with ∼25 ml of fresh HEPES bicarbonate buffer, the effect of NOS inhibition was then tested (L-NAME (100 μM)). Lumen diameter was recorded immediately and at 5 min and the percent constriction was determined from prior to L-NAME application. After another flush with HEPES buffer, the MCAs were maximally dilated at 100 mmHg with nifedipine (3 μM). Under this condition, the constriction in response to intracellular Ca^2+^ release from the sarcoplasmic reticulum was measured by addition of 1.23 × 10^−7^ M vasopressin. There is a phasic response associated with vasopressin under these conditions, which does not occur under conditions where sarcoplasmic calcium store is depleted with cyclopiazonic acid (10 μM), or calcium free 5 mM ethylene glycol-bis(β-aminoethyl ether)-N,N,N′,N′-tetraacetic acid (EGTA) Krebs. The sarcoplasmic calcium store with the MCA smooth muscle is only replenished by calcium entry through the L-type channels, which, upon blocking, demonstrates the phasic MCA contractile response corresponding to release and depletion of the sarcoplasmic calcium stores. This phasic response was recorded within the 2 min of vasopressin application. PKC activation was determined by addition of 1 μM phorbol-dibutyrate, with maximal contraction measured after 5 min of incubation. As with vasopressin, the MCAs first underwent maximal dilation at 100 mmHg using nifedipine (3 μM). Under the latter condition, phorbol dibutyrate constriction is PKC mediated and completely inhibited by PKC inhibitors chelerythrine (12 μM) or bisindolylmalemide (5 μM). The percent contraction from this maximally dilated state after nifedipine treatment was calculated after the use of phorbol dibutyrate.

### Statistical analysis

Statistical analysis was performed using SigmaPlot 12.5 (Systat Software Inc., San Jose, CA, USA) and Excel 2010 (Microsoft Corporation, Redmond, WA, USA). Data were analyzed using either one-way, or two-way ANOVA, and Holm-Sidak post-hoc analysis. Values of p < 0.05 were considered statistically significant. Except where otherwise indicated, all data are expressed as mean ± SEM.

## Results

### Effect of diet and systemic inflammation

#### Neuronal and brain damage

**Figure 1:**
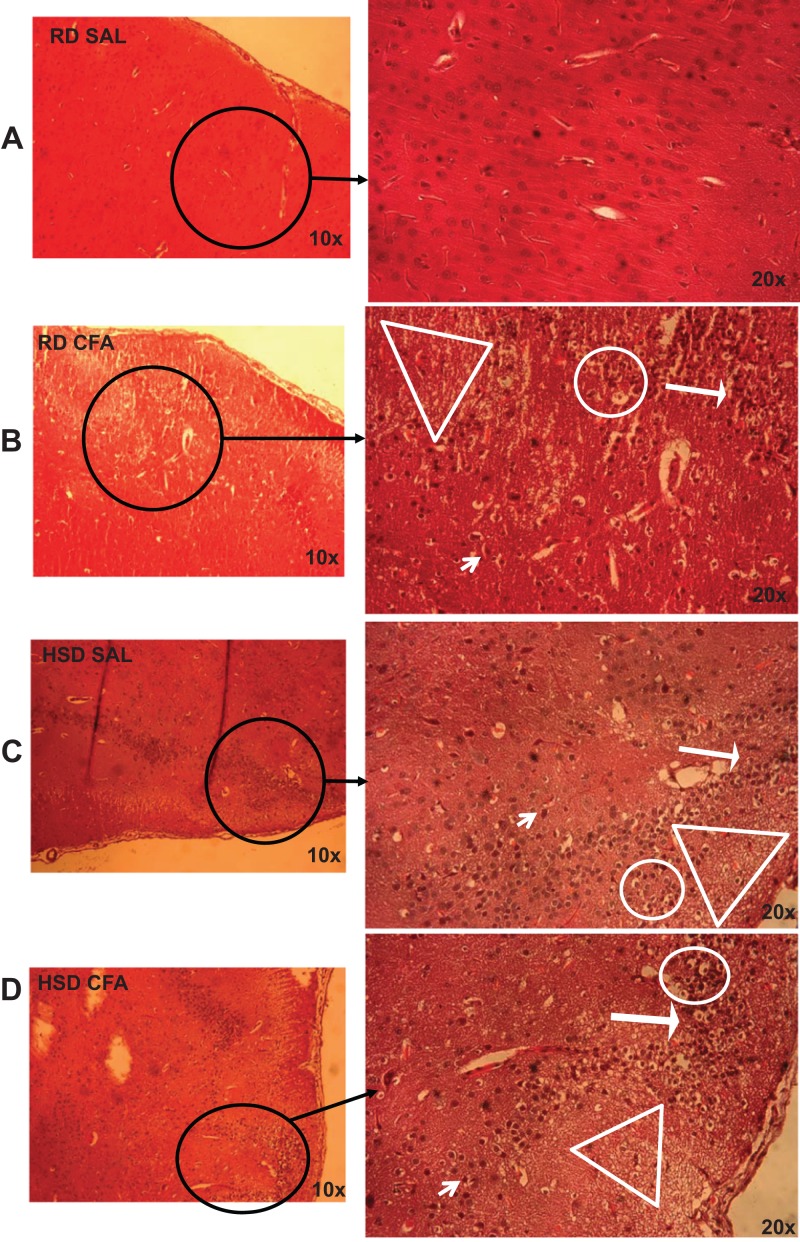
Brain pathology using H&E stains. Representative images of H&E stains of brain slices (6 µm) imaged at 10× and 20× objectives for regular diet SHRs with SAL and CFA treatments (RD SAL; A, RD CFA; B) and high salt diet SHRs with SAL and CFA treatment (HSD SAL; C, HSD CFA; D). Areas of neural cell vacuolation (circle), neural degeneration (short arrow), area of oedema (open triangle), and cell infiltration of inflammatory origin (long arrow) are indicated between treatment groups (A–D).

**Figure 2:**
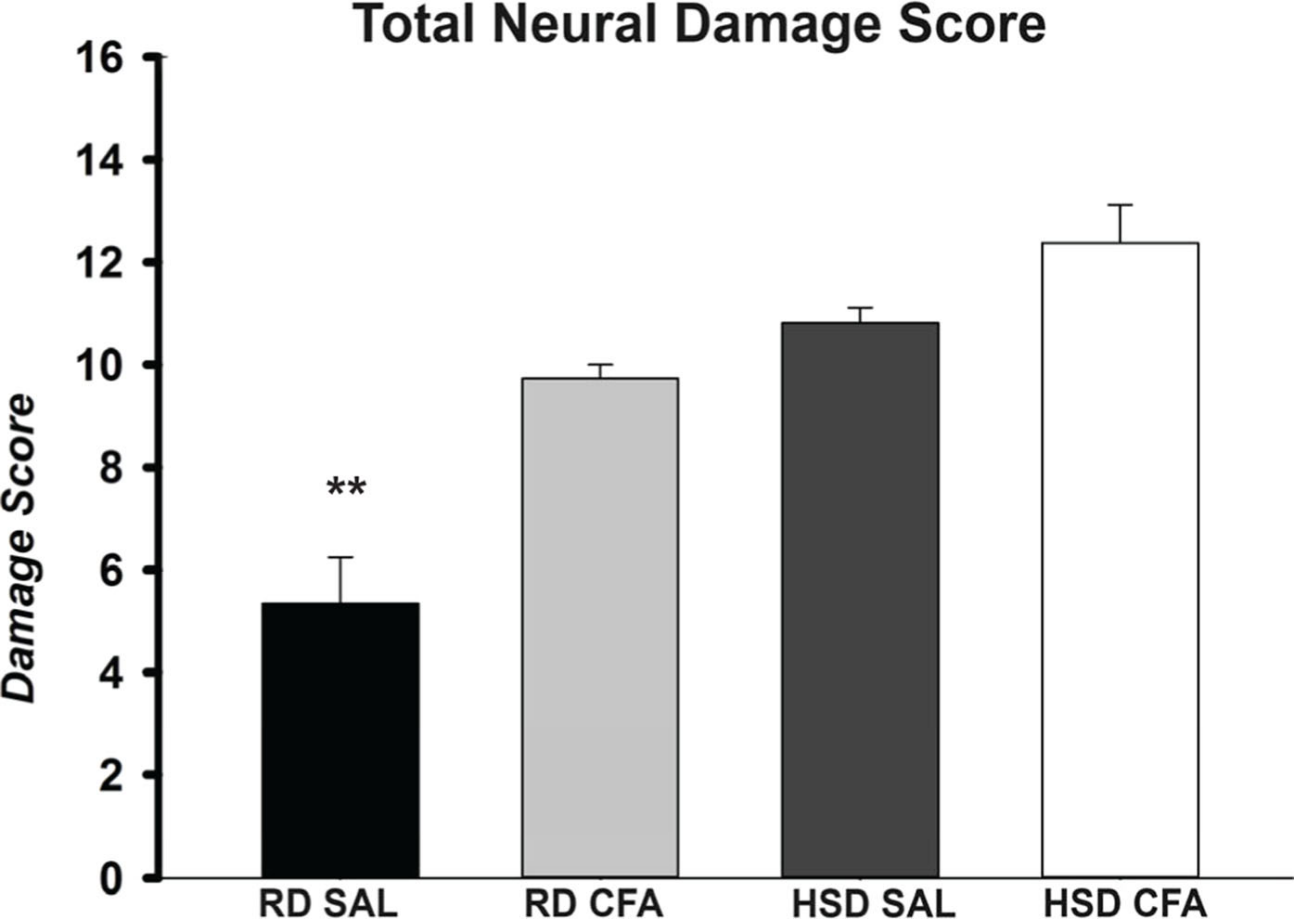
Total neuronal damage score. The H&E stained images were evaluated for the degree of neural and total brain damage of the cortex area around the middle cerebral artery. Using the scoring system indicated in Table 1, the level of damage was also scored for cell vacuolation, neuron degeneration, percent area of oedema & percent area of cell infiltration and the combined score is presented in (E). There is significant increase in neural vacuolation, degeneration, oedema and cell infiltration associated with CFA and HSD around the cortex, indicating increase in intracerebral damage associated with systemic inflammation and the high salt diet. The sample sizes ranged from n = 3–6/group. All values represent mean ± SEM. Data was analyzed using one-way ANOVA using the Holm-Sidak post-hoc test. ** p < 0.001.

H&E stains of the brains from the four experimental groups were imaged to determine degrees of intracerebral and neural damage particularly around the middle cerebral area (Figs. 1A–1D). Cell vacuolation, neuron degeneration, areas of edema, and cell infiltration appeared to increase with CFA treatment, regardless of diet. Animals on a high salt diet alone (HSD SAL) also appeared to exhibit a similar degree of neuronal damage as CFA treatment. The degree of neuronal damage was further evaluated using a four parameter semi-quantifiable scoring system (Fig. 2). Total brain damage scoring indicated a significant difference in degree of damage with induction of systemic inflammation, as well as with the HSD, compared to RD SAL control. Interestingly, out of the four parameters of brain damage, the most indicative sign of neural damage appeared to be presence of cell vacuolations (Table S1).

#### Signs of inflammatory activation: central and systemic

**Figure 3:**
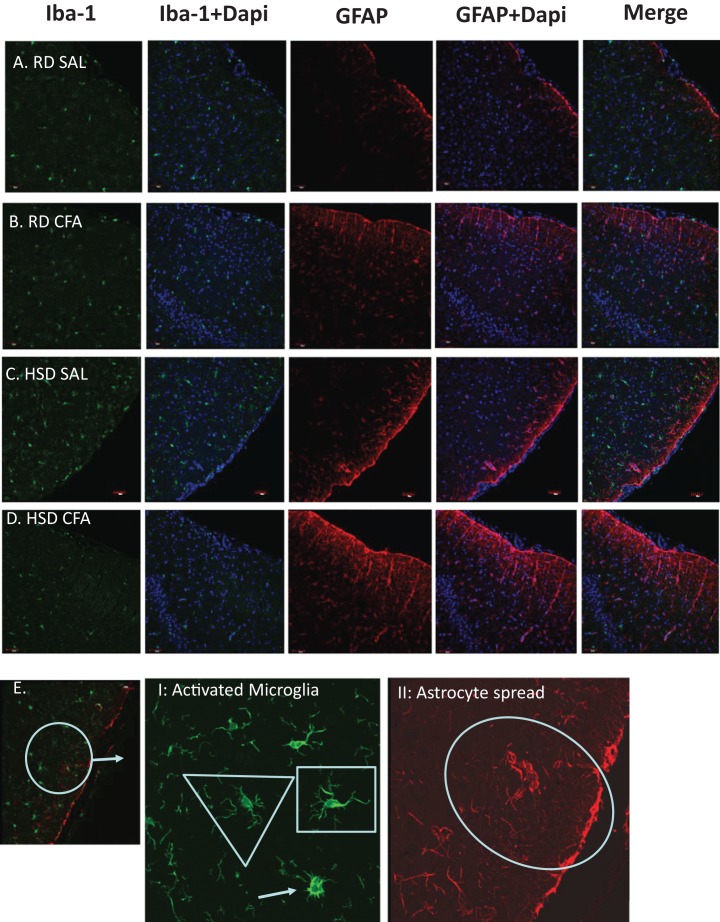
Immunostaining for astrocytes and microglia. Representative immunostaining of the cortex of the brain slices (6 µm) stained for astrocytes (GFAP- 1:1,000), microglia (Iba-1- 1:1,000) and DAPI (1:1,000) is used for nuclear staining. Staining was performed in brains of regular diet SHRs with SAL and CFA treatment (RD SAL; A, RD CFA; B) and high salt diet SHRs with SAL and CFA treatment (HSD SAL; C, HSD CFA; D). Images are at 20× objective, and scale at 100 µm for Immunofluorescence. Images were later semi-quantified for activated microglia using changes in microglia morphology (E I: Activated microglia) and area of astrocyte projection fluorescence (E II: Astrocyte spread). Triangles represent normal microglia morphology, and the arrow is the ameboid microglias which are activated. Shortened projections and bright nucleus were deemed to be microglia on the verge of full activation. Oval represent the degree of astrocyte spread from the peripheral cortex moving into the corpus colusum. Images of the DAPI staining merged with the Iba-1 stain and GFAP stain are also represented.

**Figure 4:**
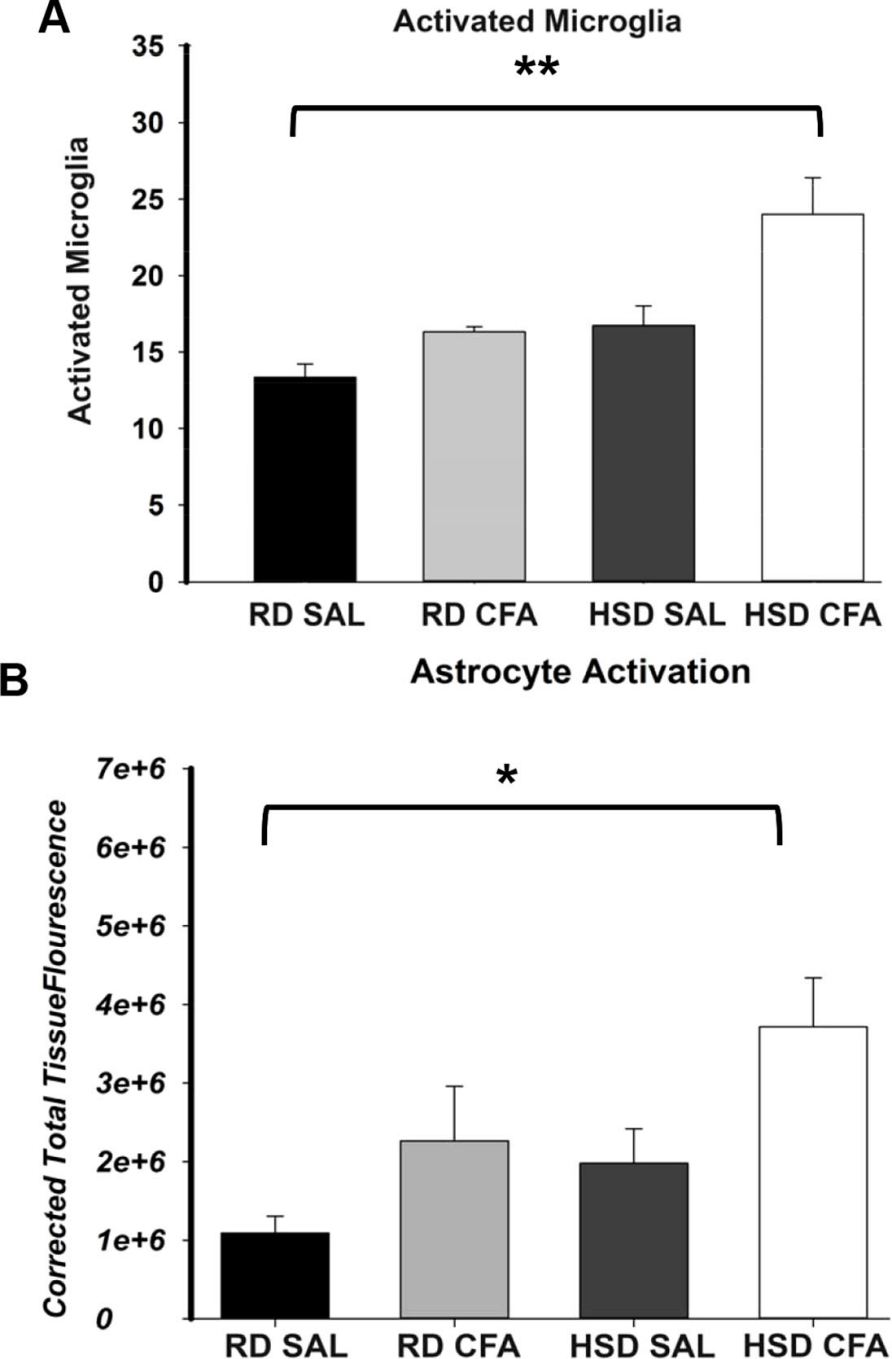
Semi-quantification of activated microglia and astrocytes in the brain. The number of activated microglia (Iba-1 staining; A) and astrocyte projection fluorescence (GFAP staining; B) in brains of regular diet SHRs with SAL and CFA treatment (RD SAL; RD CFA) and high salt diet SHRs with SAL and CFA treatment (HSD SAL; HSD CFA) were analyzed and quantified. Activated microglia was morphologically determined to be amoeboid shaped with short projections from the fluorescence images and the total detectable microglia cells overlapped with the DAPI Stained nuclei were used for the quantification analysis over a 630 µm area. The integrated density of astrocytes, presented as CTTF (Corrected total tissue fluorescence), was measured for the same area. The sample sizes ranged from n = 3–6/group. All values represent mean ± SEM. Data was analyzed using one-way ANOVA using the Holm-Sidak post-hoc test. * p < 0.05, and ** p < 0.001.

Total microglia and astrocytes were imaged using brain slices immunostained with Iba-1 and GFAP (Fig. 3). Semi-quantitative analysis of the number of activated microglia indicated a trend toward increase in activation of microglia with CFA treatment; a statistically significant increase in its activation was observed in the HSD CFA compared to the RD SAL group (Fig. 4A). Quantification of the integrated density of GFAP staining determined astrocyte spread from the cerebral neocortex towards the corpus callosum. A numerical trend toward an increase in integrated density in the RD-CFA compared to RD SAL was also observed (Fig. 4B). HSD appeared to increase both astrocyte density and its spread from the neocortex, as well as the expression of activated microglia. However, no statistical significance was observed. CFA treatment in the HSD-CFAs caused a significant increase in astrocyte density compared to RD SAL, indicating an increase in neuro-inflammatory reaction, likely associated with CFA injection and HSD combined. The resulting increase in neuro-inflammation may contribute to the degree of severity of damage in the brain with CFA treatment, exacerbated by HSD, as previously quantified in this animal model. An example of the hemorrhages observed with CFA treatment is shown in Fig. S1. In order to determine whether we also observed other organ damage associated with the chronic inflammation, we analyzed the kidneys for presence of proteinaceous casts and inflammatory infiltrate related to both diet and inflammatory injury (Fig. S2). We found RD SAL have normal glomeruli, with little to no proteinaceous casts within the cortex and medulla. In comparison, the RD CFA have occasional proteinaceous casts in the medulla, as do the HSD SAL group. Interestingly, the HSD SALs also express minimal inflammatory infiltrate and occasional obsolete glomerulus. In contrast, the HSD CFA express abundant protein casts, abundant obsolete glomeruli, severe glomerular sclerosis, and prominent inflammatory infiltrate around the glomeruli and blood vessels.

### Effect of diet and inflammation on vascular function in the MCA

#### Pressure dependent constriction

**Figure 5:**
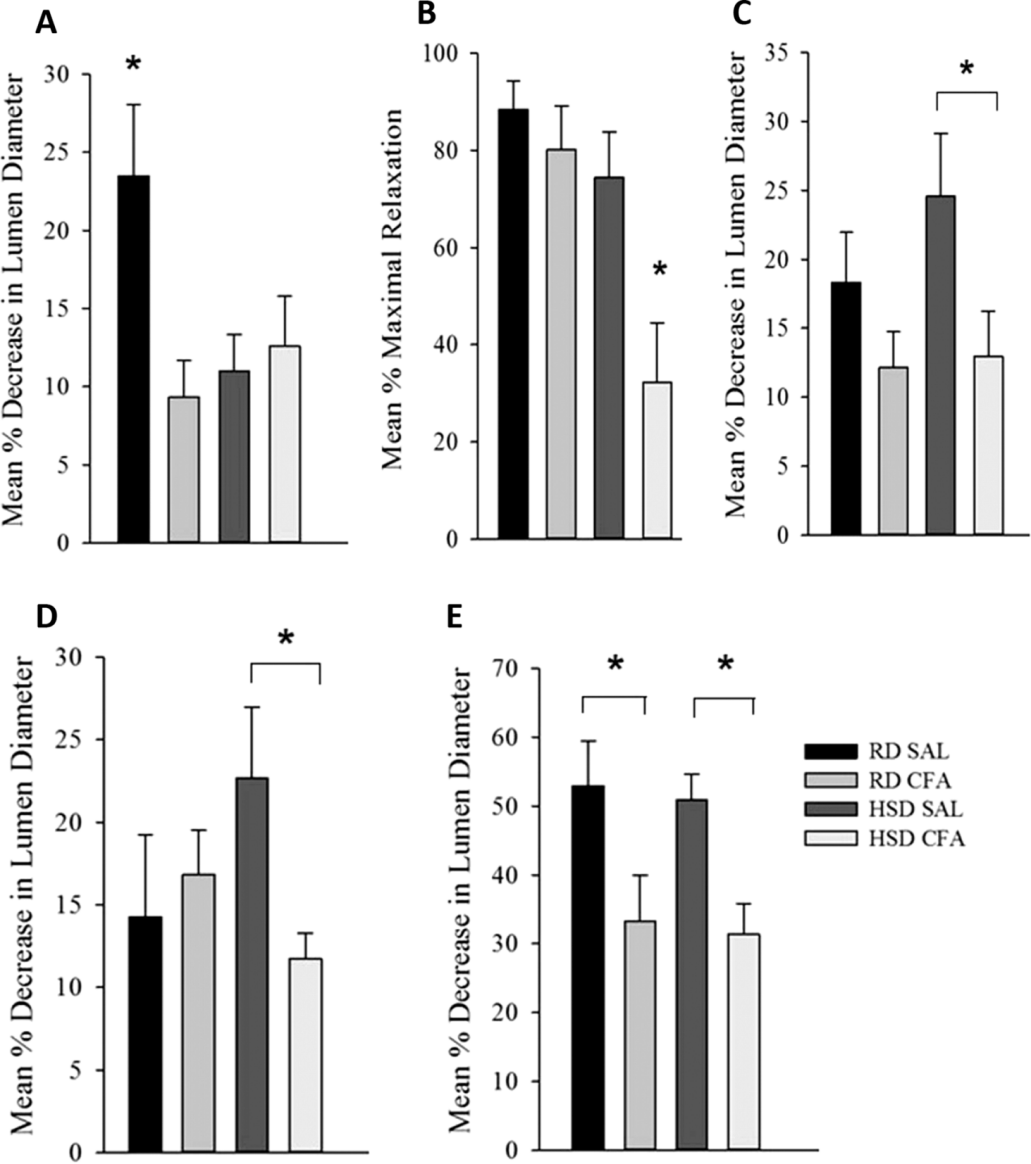
Effect of complete Freund’s Adjuvant and/or high salt diet on middle cerebral artery function. Pressure myograph studies were performed in the MCAs isolated from RD SAL (n = 9–12), RD CFA (n = 10–13), HSD SAL (n = 11–13), HSD CFA (n = 9–16). The ability to respond to pressure step (PDC; A) showed RD SAL maintained normal PDC response. There was a significant decrease in PDC in RD CFA vs. RD SAL. No difference was observed between HSD CFA vs. HSD SAL. HSD itself significantly diminished capacity for MCA’s ability to undergo PDC HSD SAL vs. RD SAL vs. HSD SAL. Bradykinin response (B) show no difference in bradykinin-induced vasodilation between RD CFA vs. RD SAL. However, endothelial vasodilatory response was significantly diminished in HSD CFA vs. HSD SAL. There was no significant difference between HSD SAL vs. RD SAL. L-NAME response (C) indicate a trend toward diminished L-NAME response is seen in RD CFA vs. RD SAL, but it was not significant. The HSD CFA response to L-NAME was, however, significantly lower than that of HSD SAL. HSD did not affect L-NAME response vs. RD (HSD SAL vs. RD SAL). Constriction response to Vasopressin (D) show no significant difference between RD CFA vs. RD SAL. However, inflammation induced a significant decrease in response to vasopressin in HSD CFA vs. HSD SAL. No difference was observed to vasopressin due to diet (HSD SAL vs. RD SAL). Response to phorbol dibutyrate (E), indicate induction of inflammation significantly diminishes response to PKC activation regardless of diet (RD CFA vs. RD SAL and HSD CFA vs. HSD SAL). There were no diet-induced differences in response to phorbol dibutyrate. All values represent mean ± SEM. Data was analyzed using two-way ANOVA using the Holm-Sidak post-hoc test. * p < 0.05.

Both diet and CFA treatment affected PDC (Fig. 5A). CFA treatment appeared to significantly diminish the ability of MCAs to constrict to pressure in the RD group compared to SAL controls. However, there was no significant difference in MCA vasoconstriction in HSD CFA compared to HSD SAL, indicating that HSD alone had greatly diminished the vessel’s ability to respond to luminal pressure. This is further evidenced by a significant decrease in PDC response in the HSD SAL group compared to the RD SAL group (p = 0.01).

#### Endothelial function: response to bradykinin

Figure 5B depicts the endothelial vasodilatory response of MCA’s to addition of bradykinin (1.6 μM). The effect of CFA was not evident within MCAs of RD groups (RD CFA vs. RD SAL). However, a statistically significant decrease in response was observed in the MCA’s from HSD-fed CFA rats compared to HSD SAL rats (p = 0.015). There was no difference in vessel response to bradykinin due to the HSD (i.e. RD SAL vs. HSD SAL). However, comparison between inflamed groups of the different diets indicated a significant decrease in relaxation in the HSD CFA cohort compared to the RD CFA (p = 0.006), demonstrating the combined effect of both HSD and inflammation on bradykinin response in the MCAs.

#### Endothelial function: NOS function

Endothelial-mediated relaxation by nitric oxide (NO) was tested by the addition of a non-specific NOS inhibitor L-NAME (100 μM), eliminating NO-mediated vasodilation (Fig. 5C). Induction of inflammation via CFA did not significantly decrease response to L-NAME in the RD groups despite a trend in depressed response (RD CFA vs. RD SAL). However, there was a statistically significant decrease observed with CFA treatment in the HSD groups (HSD CFA vs. HSD SAL; p = 0.018). No statistically significant difference was noted in MCA response to L-NAME between diets (RD-SAL vs. HSD-SAL).

#### Intracellular calcium response

Intracellular Ca^2+^ release was evaluated in the presence of nifedipine (L-type calcium channel blocker; 3 μM) and analyzing the MCA’s response to intracellular Ca^2+^ release by vasopressin (1.23 × 10^−7^M). There was no significant difference in the treatments in the RD group in their response to sarcoplasmic calcium release (RD CFA vs. RD SAL). However, in the HSD group, a statistically significant difference was observed between inflamed and non-inflamed rats, as the MCAs of HSD CFA group had a significant diminished response to vasopressin compared to the HSD SAL group (p = 0.03) (Fig. 5D). There was no difference in vessel contraction in response to vasopressin between diets (RD SAL vs. HSD SAL).

#### PKC activation

Phorbol Dibutyrate (1 μM) was added to the MCAs to evaluate vascular smooth muscle response to PKC activation in the presence of nifedipine (3 μM) (Fig. 5F). A significant difference was observed in the inflamed (CFA) groups compared to SAL in both RD and HSD groups (p = 0.047, RD CFA vs. RD SAL; p = 0.018, HSD CFA vs. HSD SAL). There was no statistical difference in response to PKC activation between the diets (RD SAL vs. HSD SAL).

## Discussion

The arthritic hypertensive model exemplifies a moderate arthritic response localized in one paw, which induces systemic inflammation and also maintains high systolic blood pressure independent of diet or inflammatory treatment as previously published by our group (Randell & Daneshtalab, 2016). The joint damage with our mono-arthritis model is reminiscent of the changes that occur with RA (Kannan, Ortmann & Kimpel, 2005) with increases in systemic inflammatory mediator Tumor Necrosis Factor alpha (TNF-α) (Randell & Daneshtalab, 2016). Uniquely, we had also found an associated incidence of HS alongside increase in systemic inflammatory injury. In this study, we have determined there is loss of the ability of the MCA to undergo PDC and respond to vasogenic drugs, which likely contribute to incidence of intracerebral hemorrhage in this model. These differences are either dependent on chronic inflammation (CFA injection), diet (HSD or RD) or both.

We have previously shown that there is an increase in systemic TNF-α in the HSD-CFA groups (Randell & Daneshtalab, 2016). TNF-α directly affects joint degeneration and destruction in arthritis (Saklatvala, 1986), and induces a cascade of other pro-inflammatory cytokines and proteins such as interleukins, prostaglandins, and angiotensin II in peripheral organs (Brennan & Feldmann, 1992; Feldmann & Maini, 2008). H&E staining within the cortex of the brain show evidence of an increase in axonal and nerve cell damage indicated by neural vacuolation, nerve degeneration, edema, and cell infiltrates with CFA treatment (Figs. 1 and 2). Astrocyte branching within the brain also appear to increase, spreading inward from the cortex into the grey matter with inflammatory stimulus. Central inflammation is also apparent with increases in activated microglia associated with the systemic inflammatory injury (Figs. 3 and 4). Our observations lead us to believe the central inflammatory response and neuronal damage is associated with the increase in pro-inflammatory levels with systemic inflammation. The pro-inflammatory mediators may also increase the nephro-glomerular damage in the kidneys (also observed in our animal model) which in turn increase urea and uric acid, weakening the blood brain barrier (BBB) and increasing toxicity and neural inflammatory response (Henke et al., 2007; Izawa-Ishizawa et al., 2012).

HSD itself appears to cause neural inflammation, damage, and increased immune activation in both kidneys and the brain; (Figs. 1–4 and S2). Slices of brain cortex indicate HSD-driven increases in astrocytes branching and expression, as well as numerical increases in activated microglia staining (Fig. 4). The role of sodium driving autoimmune diseases has been presented by various groups in the last couple of years, with sodium chloride activating inflammatory pathways (Croxford, Waisman & Becher, 2013; Kleinewietfeld et al., 2013). Our model clearly indicates that the addition of inflammatory insult to the HSD exacerbates the inflammatory response, and likely increases the severity of the cerebral hemorrhage that had been observed in the HSD CFA rats.

When we examine the MCA’s ability to undergo PDC, we find that the loss of MCA function is linked to spontaneous HS development in the SHRsp model. We have previously shown loss of MCA function in the SHRsps contributed to the inability to undergo PDC and autoregulation in the brain (Smeda & Daneshtalab, 2011). The loss of response to intraluminal pressure in the HSD SAL rats is likely attributed to the effects of both inflammation and chronic HSD on the endothelium. Endothelial dysfunction secondary to chronic salt intake has been linked to increased endothelial production of factors that increase the production of reactive oxygen species (ROS) (Durand et al., 2010; Feng et al., 2015). Significantly diminished MCA function due to the high salt may have decreased the endothelial function such that inflammatory insult via CFA was negligible in the HSD CFA group. The direct effect of inflammatory insult on MCA function is observed in our RD CFA groups, as the MCAs did not contract significantly to high luminal pressure. Both the endothelium and vascular smooth muscle cell dysfunction may have occurred due to the trigger of physical and chemical stress signals (Numata, Takahashi & Inoue, 2015) and kinases such as NF-κB (Chauhan et al., 2014). The trigger may affect specific endothelial transient receptor potential (TRP) channels such as TRPV1 and TRPV4 with subsequent vasodilation (Kwan, Huang & Yao, 2007), thus impairing pressure-induced contractile response in RD CFAs while maintaining bradykinin’s endothelial response.

The loss of NO release and altered regulation in the endothelium can be exacerbated by chronic high salt and inflammatory insult together, seen in HSD CFAs. The detrimental effect of pro-inflammatory mediators on the endothelial response likely occurs through decrease in regulation of endothelial nitric oxide (eNOS) and endothelial derived hyperpolarizing factor (EDHF; Neumann, Gertzberg & Johnson, 2004)–potentially activated by bradykinin (Félétou & Vanhoutte, 2009), leading to diminished EDHF-initiated relaxation of the vascular smooth muscle (Kessler et al., 1999). The lack of significant difference in L-NAME or bradykinin response between inflamed and non-inflamed RD-fed SHR may be due to a lower TNF-α response seen in the RD CFA rats compared to RD SAL rats (Randell & Daneshtalab, 2016).

A significant degree of vascular smooth muscle dysfunction was also observed, associated with both diet and inflammation. While the HSD CFA group had a significantly diminished response to both vasopressin and phorbol ester (compared to HSD SAL), the RD CFA group only showed a diminished response to PKC activation (compared to RD SAL; Figs. 5D and 5E). The impaired response to PKC activation in both inflamed groups is akin to the decrease in PKC activity previously observed in the post-stroke SHRsp (Smeda, King & Harder, 1999). MCA remodeling due to pro-inflammatory mediators may account for the dysfunctional response to sarcoplasmic calcium release (vasopressin) as well as PKC activation (phorbol dibutyrate) (Bastin & Heximer, 2011). Also, there is evidence of increasing matrix metalloproteinase activity and increasing proliferation of vascular smooth muscle cells with TNF-α (Lee, Kim & Moon, 2009; Pires et al., 2014). The combined effect of diet and inflammatory insult on V1 receptor (and activation of the PLC/IP_3_ system) in our HSD CFA group may have led to the failed response to vasopressin as well, while CFA treatment alone may only affect PKC activation via DAG/PKC pathway; PKC, which requires Ca^2+^ and DAG for activation, has several downstream effects. It is known to interact with MLCK, ERK1/2, Rho kinase and calmodulin-dependent protein kinase II in addition to membrane channels to elicit vascular smooth muscle contraction (Babwah, Dale & Ferguson, 2003; Bastin & Heximer, 2011). Interestingly, the RD CFA group still show pinpoint hemorrhages despite a functioning MCA (Fig. S1) indicating it is still possible to have intracerebral hemorrhage without exhibiting all levels of dysfunction exemplified by the post-stroke SHRsp. The exact signaling alterations associated with the MCA dysfunction remains to be determined.

## Conclusions

Our results indicate that inflammatory injury in the setting of high dietary sodium intake and chronic hypertension leads to a more severe course of inflammatory autoimmune disease. It predisposes the patient to developing an apparently more severe form of HS, organ damage, neuro-inflammation, and loss of cerebrovascular response. The physiological mechanism leading to intracerebral hemorrhage and neural damage is multi-factorial and includes severe hypertension, vascular dysfunction leading to loss of autoregulation of cerebral blood flow, weakening of the BBB leading to cerebral hemorrhage formation (Yamori & Horie, 1977). It is likely that elevated dietary sodium alone is initiating a type of inflammatory process and evidence of cerebral damage. Subsequent inflammatory insult by way of CFA injection inarguably exacerbates this process. The varying levels of neural damage observed in our model, being less severe than what is usually observed in the SHRsp model, allows us to correlate the subsequent tiers of cerebrovascular dysfunction. We believe that patients suffering from RA (and similar systemic inflammatory process) likely experience changes to the cerebral vessels and experience similar neural inflammatory damage, exacerbated by the HSD, as exemplified by our hypertensive-arthritic rat model. Further investigation into the precise mechanisms of neural damage incurred in the RA population suffering from hypertension will promote application of patient-specific treatments to prevent increase in mortality.

## Supplemental Information

10.7717/peerj.2608/supp-1 Supplemental Information 1 Raw data files for the myogenic contraction experiments.

10.7717/peerj.2608/supp-2 Supplemental Information 2 Gros cerebral damage induced by Complete Freund’s Adjuvant and/or high salt diet treatment. Brains of RD CFA (A) and HSD CFA (B) (n = 5) infused with Evans Blue Dye (15 mg/kg). None of the brains of RD SAL or HSD SAL (n = 3/group) showed any dye extravascation or brain abnormalities which include septum deviation, decreased perfusion of the brain, edemic brain, or difference in hemisphere size. Almost 70% of the HSD CFA groups (infused or not infused with Evans Blue Dye) exhibited other symptoms of brain abnormalities often associated with hemorrhagic stroke, albeit in a lower degree than the stroke prone SHR strain. They include a significantly diminished brain perfusion, edema of the brain (watery brain), septum deviation. The regular diet CFA groups in comparison exhibited increase in pain point hemorrhages (evidenced only by the Evans Blue Dye extravasation). However, the brain morphology in general was more healthy.

10.7717/peerj.2608/supp-3 Supplemental Information 3 Kidney pathology associated with the hypertensive arthritic rat model. To assess protein cast formation and glomerular injury, renal tissue sections (4 μm) were stained with Periodic Acid Schiff (PAS) stain and imaged using a light microscope (Olympus). Representative micrographs are displayed to indicate renal pathology. PAS staining illustrated that the regular diet group treated with saline only (RD Sal) have normal glomeruli, with little to no proteinaceous casts within the cortex and medulla (A). In contrast, the regular diet group induced with CFA (RD CFA; B) have occasional proteinaceous casts (arrowhead) in the medulla while the glomeruli appear normal. In the high salt diet group treated with saline (HSD SAL; C) proteinaceous cast appear within the cortex and the medulla (arrowheads). This is accompanied by an inflammatory infiltrate (arrow) with occasional obsolete glomerulis (*). The high salt diet group treated with CFA (HSD CFA; D) express abundant protein casts throughout the medulla and the cortex (arrowheads), abundant obsolete glomerulus (*), severe glomeruli sclerosis, and prominent inflammatory infiltrate around the glomeruli and blood vessels (arrow). Overview: Bar = 500 μm; Detail: Bar = 100 μm.

10.7717/peerj.2608/supp-4 Supplemental Information 4 Raw data for the four parameters determining brain damage. Table S1: The four semi-quantified parameters for cell vacuolation, degenerating neurons, area of cell oedema and area of cell infiltration were calculated. We used H&E stain images presented in Fig. 2 for regular diet SHRs with and without CFA (RD SAL; RD CFA), and for high salt diet SHRs with and without inflammation (HSD SAL; HSD CFA). The parameters for brain damage outlined in Table 1 and in Materials and Methods section. Cell vacuolation and neuron degeneration are two important parameters that can be measured by H&E stain for marking the cell death. The area of oedema and area of cell infiltration, quantified as percentage, used the complete image area presented at 200× magnification. Oedema and cell infiltration are indicators of brain damage. Data was analyzed with n = 3−6/group. The values are presented as average ± SEM. Data was analyzed using one way ANOVA and Holm-Sidak post hoc analysis. ** indicates p < 0.001 from RD Sal in their respective parameters.

## List of Abbreviations

CV: Cardiovascular
CFA: Complete Freund’s Adjuvant
HS: Hemorrhagic stroke
HSD: High salt diet
L-NAME: L-N^G^-Nitroarginine methyl ester
MCA: Middle cerebral artery
NOS: Nitric oxide synthase
PDC: Dependent constriction
RA: Rheumatoid Arthritis
SAL: Saline
SHR: Spontaneously hypertensive rat
SHRsp: Stroke prone spontaneously hypertensive rat
PKC: Protein kinase C
RD: Regular diet
TRP: Transient receptor potential.

## Additional Information and Declarations

### Data Deposition

The following information was supplied regarding data availability: The raw data has been supplied as Supplemental Dataset Files.
