# Supplementary figures and images for: Alterations to the middle cerebral artery of the hypertensive-arthritic rat model potentiates intracerebral hemorrhage
Source: PeerJ. 2016 Nov 3;4:e2608. doi: 10.7717/peerj.2608 (PMC5101607; doi:10.7717/peerj.2608)

**A. RD CFA**

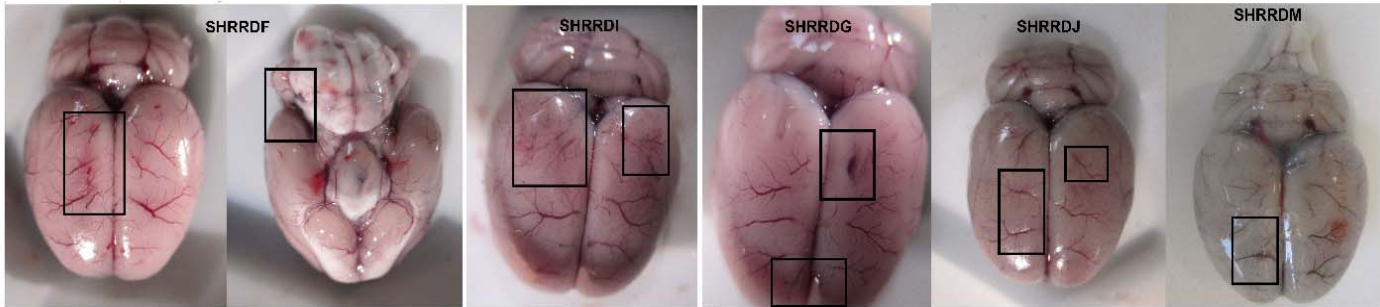

**B. HSD CFA**

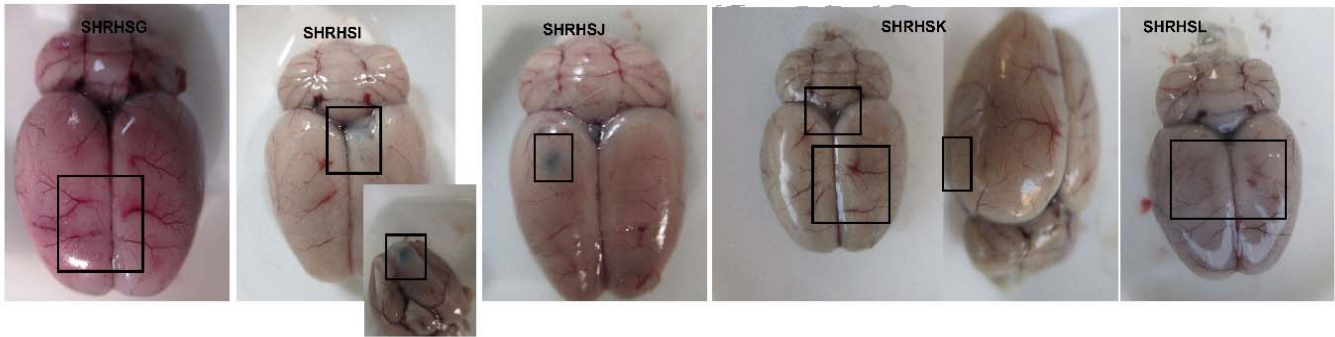

Supplement: Supplemental Information 2 — Brains of RD CFA (A) and HSD CFA (B) (n = 5) infused with Evans Blue Dye (15 mg/kg). None of the brains of RD SAL or HSD SAL (n = 3/group) showed any dye extravascation or brain abnormalities which include septum deviation, decreased perfusion of the brain, edemic brain, or difference in hemisphere size. Almost 70% of the HSD CFA groups (infused or not infused with Evans Blue Dye) exhibited other symptoms of brain abnormalities often associated with hemorrhagic stroke, albeit in a lower degree than the stroke prone SHR strain. They include a significantly diminished brain perfusion, edema of the brain (watery brain), septum deviation. The regular diet CFA groups in comparison exhibited increase in pain point hemorrhages (evidenced only by the Evans Blue Dye extravasation). However, the brain morphology in general was more healthy. [file peerj-04-2608-s002.pdf]

## Overview

## Detail

**A**

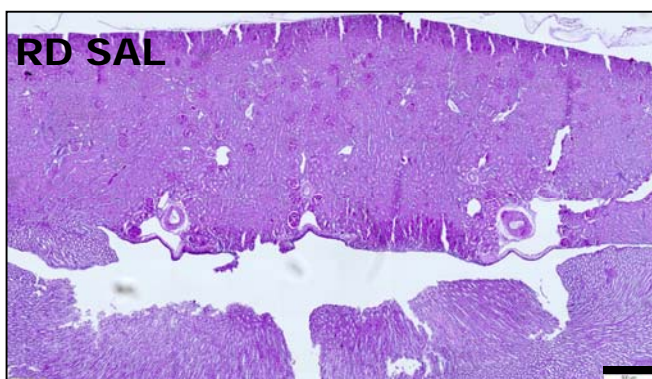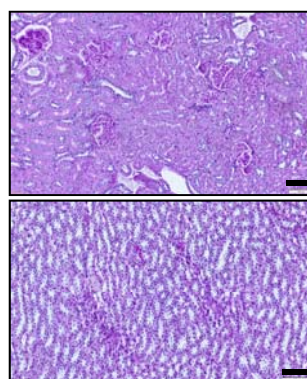

**B**

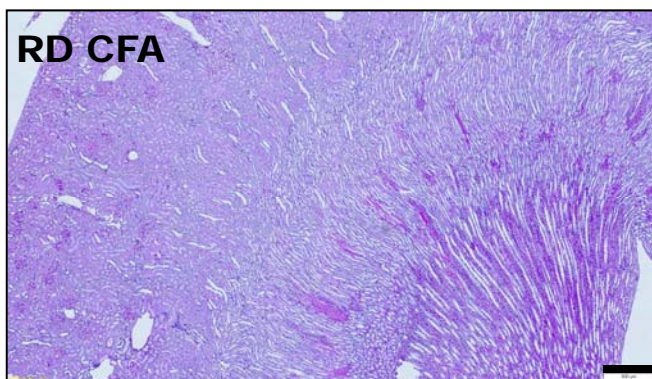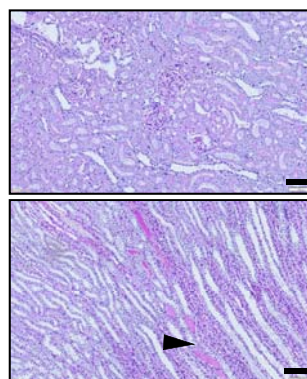

**C**

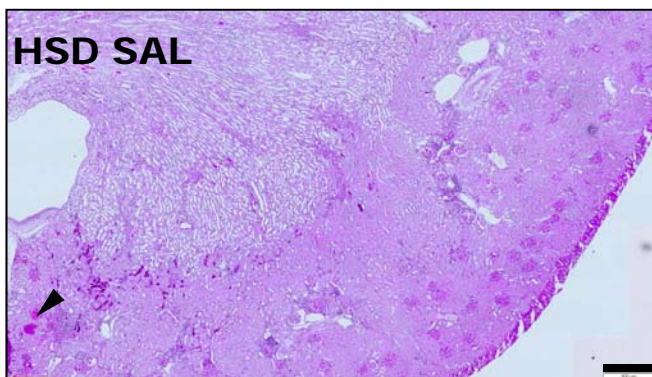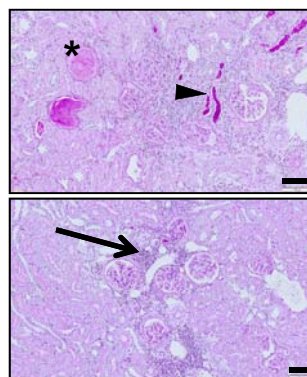

**D**

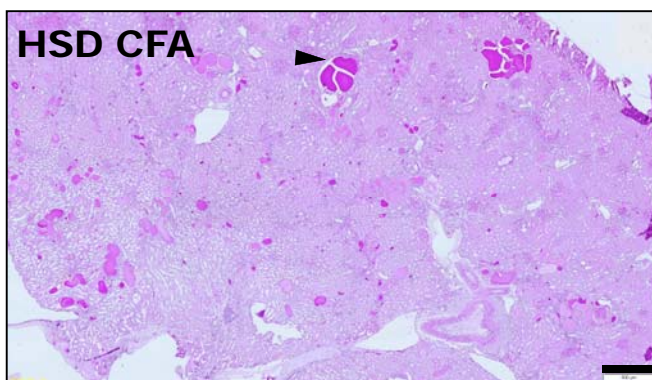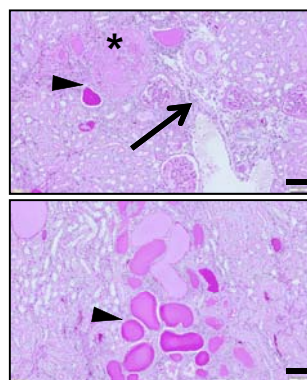

Supplement: Supplemental Information 3 — To assess protein cast formation and glomerular injury, renal tissue sections (4 μm) were stained with Periodic Acid Schiff (PAS) stain and imaged using a light microscope (Olympus). Representative micrographs are displayed to indicate renal pathology. PAS staining illustrated that the regular diet group treated with saline only (RD Sal) have normal glomeruli, with little to no proteinaceous casts within the cortex and medulla (A). In contrast, the regular diet group induced with CFA (RD CFA; B) have occasional proteinaceous casts (arrowhead) in the medulla while the glomeruli appear normal. In the high salt diet group treated with saline (HSD SAL; C) proteinaceous cast appear within the cortex and the medulla (arrowheads). This is accompanied by an inflammatory infiltrate (arrow) with occasional obsolete glomerulis (*). The high salt diet group treated with CFA (HSD CFA; D) express abundant protein casts throughout the medulla and the cortex (arrowheads), abundant obsolete glomerulus (*), severe glomeruli sclerosis, and prominent inflammatory infiltrate around the glomeruli and blood vessels (arrow). Overview: Bar = 500 μm; Detail: Bar = 100 μm. [file peerj-04-2608-s003.pdf]
