# Supplementary material for: Alterations to the middle cerebral artery of the hypertensive-arthritic rat model potentiates intracerebral hemorrhage
Source: PeerJ. 2016 Nov 3;4:e2608. doi: 10.7717/peerj.2608 (PMC5101607; doi:10.7717/peerj.2608)
Supplement: Supplemental Information 4 — Table S1: The four semi-quantified parameters for cell vacuolation, degenerating neurons, area of cell oedema and area of cell infiltration were calculated. We used H&E stain images presented in Fig. 2 for regular diet SHRs with and without CFA (RD SAL; RD CFA), and for high salt diet SHRs with and without inflammation (HSD SAL; HSD CFA). The parameters for brain damage outlined in Table 1 and in Materials and Methods section. Cell vacuolation and neuron degeneration are two important parameters that can be measured by H&E stain for marking the cell death. The area of oedema and area of cell infiltration, quantified as percentage, used the complete image area presented at 200× magnification. Oedema and cell infiltration are indicators of brain damage. Data was analyzed with n = 3−6/group. The values are presented as average ± SEM. Data was analyzed using one way ANOVA and Holm-Sidak post hoc analysis. ** indicates p < 0.001 from RD Sal in their respective parameters. [file peerj-04-2608-s004.docx]

| *Raw Values* | *Cells undergoing vacuolation* | *Degenerating neurons* | *Percentage of area of oedema* | *Percentage of area of Cell infiltration* |
| --- | --- | --- | --- | --- |
| RD Sal | 22 ± 3 | 1 ± 0 | 4 ± 3 | 10 ± 5 |
| RD CFA | ******42 ± 4 | 3 ± 1 | 11 ± 5 | 11± 5 |
| HSD Sal | ******43 ± 4 | ******5 ± 1 | 12 ± 3 | 17 ± 1 |
| HSD CFA | ******51 ± 6 | 3 ± 0 | 18 ± 8 | 18 ± 2 |
